# Flow invariants in a channel obstructed by a line of inclined rods

**DOI:** 10.1038/s41598-022-10204-0

**Published:** 2022-04-13

**Authors:** V. A. Herrero, H. Ferrari, R. Marino, A. Clausse

**Affiliations:** 1 Facultad de Ingeniería, LIDTUA-CIC, Universidad Austral, Pilar, Argentina; 2 Facultad de Ciencias Exactas y Naturales, Universidad Nacional de Cuyo, Mendoza, Argentina; 3 CNEA and Universidad Nacional del Centro, Tandil, Argentina; 4 CONICET, Consejo Nacional de Investigaciones Científicas y Técnicas, Ciudad de Buenos Aires, Argentina

**Keywords:** Engineering, Mechanical engineering

## Abstract

An experiment is conducted in a rectangular channel obstructed by a transverse line of four inclined cylindrical rods. The pressure on the surface of a central rod and the pressure drop through the channel are measured varying the inclination angle of the rods. Three assemblies of rods with different diameters are tested. The measurements were analyzed applying momentum conservation principles and semi-empirical considerations. Several invariant dimensionless groups of parameters relating the pressure at key locations of the system with characteristic dimensions of the rods are produced. It was found that the independence principle holds for most of the Euler numbers characterizing the pressure at different locations, that is, the group is independent of the inclination angle provided that the inlet velocity projection normal to the rods is used to non-dimensionalize the pressure. The resulting semi-empirical correlations can be useful for designing similar hydraulic units.

## Introduction

Many heat and mass transfer devices are composed by sets of modules, channels or cells through which a fluid passes amid more or less complex internal structures, like rods, buffers, inserts, etc. Recently there has been a renewed interest for a deeper understanding of the mechanisms relating the internal pressure distribution and forces on complex internals with the overall pressure drop of the modules. This interest is brought about, amongst others, by innovations in material science, expansion of computational capacity for numerical simulations, and the increasing miniaturization of devices. Recent experimental studies of pressure internal distributions and losses include channels roughened by variously shaped ribs^1^, electrochemical reactors cells^2^, capillary contractions^3^ and lattice-frame materials^4^.

The most common internal structures are arguably cylindrical rods passing through the unit module, either as bundles or isolated. In heat exchangers this configuration is typical on the shell side. Shell-side pressure drops are relevant in the design of heat exchangers such as steam generators, condensers and evaporators. In a recent study, Wang et al.^5^ found reattachment and co-shedding flow regimes in tandem configuration of rods. Liu et al*.*^6^ measured the pressure drop in a rectangular channel with a built-in double-U-shaped tube bundle with different inclinations and calibrate a numerical model emulating the rod bundle with a porous medium.

As expected, there are numerous configuration factors that influence the hydraulic performance of cylinder banks: type of arrangement (*e.g.,* staggered or in-line arrays), relative dimensions (*e.g.,* spacing, diameter, length), and inclination angle, among others. Several authors focused their efforts in finding dimensionless criteria to guide the design capturing the combining effects of the geometric parameters. Among the more recent experimental studies, Kim et al*.*^7^ proposed a model of effective porosity taking the length of the unit cell as the control parameter using in-line and staggered arrays and Reynolds numbers between 10^3^ and 10^4^. Snarski^8^ studied how the power spectra from accelerometers and hydrophones attached to cylinders in a water tunnel varied with the inclination respect to the flow direction. Marino et al*.*^9^ studied the wall pressure profile around cylindrical rods in yawed gas flow. Mityakov et al*.*^10^ mapped the velocity field in the wake of a yawed cylinder using stereo-PIV. Alam et al*.*^11^ presented a comprehensive study of tandem cylinders, focusing on the effects of the Reynolds number and geometric ratios on vortex shedding. They were able to identify five regimes, namely lock-in, intermittent lock-in, no lock-in, subharmonic lock-in and shear-layer reattachment regimes. Recent numerical studies remarked the formation of vortex structures in a flow passing confined yawed cylinders^12^.

Generally, the hydraulic performance of unit cells is expected to depend on the configuration and geometry of the internal structures, often quantified through empirical correlations that spring from specific experimental measurements. In many of these devices consisting of periodic assemblies, the flow patterns repeat in each unit cell, hence, the information related to a representative unit cell can be utilized to express the overall hydraulic behavior of the structures by means of multiscale models^13^^14^. In these symmetric cases, it is often possible to reduce the degree of specificity applying general conservation principles. A typical example is the discharge equation of orifice plates^15^. In the particular case of inclined rods, either in confined or open flows, there is an interesting criterion often cited in the literature and used by designers, which associates the main hydraulic magnitudes (*e.g.*, pressure drops, forces, vortex shedding frequency, etc.) to the component of the flow perpendicular to the axis of the cylinders. This is often referred to as the independence principle and assumes that the flow dynamics is mostly driven by the inflow normal component, and that the axial component aligned with the cylinder axis has a negligible impact. Although no consensus exists in the literature regarding the range of validity of this criterion, in many cases it provides useful estimations within the experimental uncertainties typical of empirical correlations. Recent studies of the validity of the independence principle include vortex-induced vibrations^16^ and single and two-phase average drag^4^^17^.

In the present work, the results of a study of the internal pressures and the pressure drop in a channel with a transverse line of four inclined cylindrical rods are presented. Three rod assemblies having different diameters are measured, varying the inclination angle. The general goal is to study the mechanisms relating the pressure distribution on the rods surface with the overall pressure drop of the channel. The experimental data is analyzed applying the Bernoulli equation and momentum conservation principles, to assess the validity of the independence principle. Finally, dimensionless semi-empirical correlations are produced that can be useful for designing similar hydraulic units.

## Experimental setup and method

**Figure 1:**
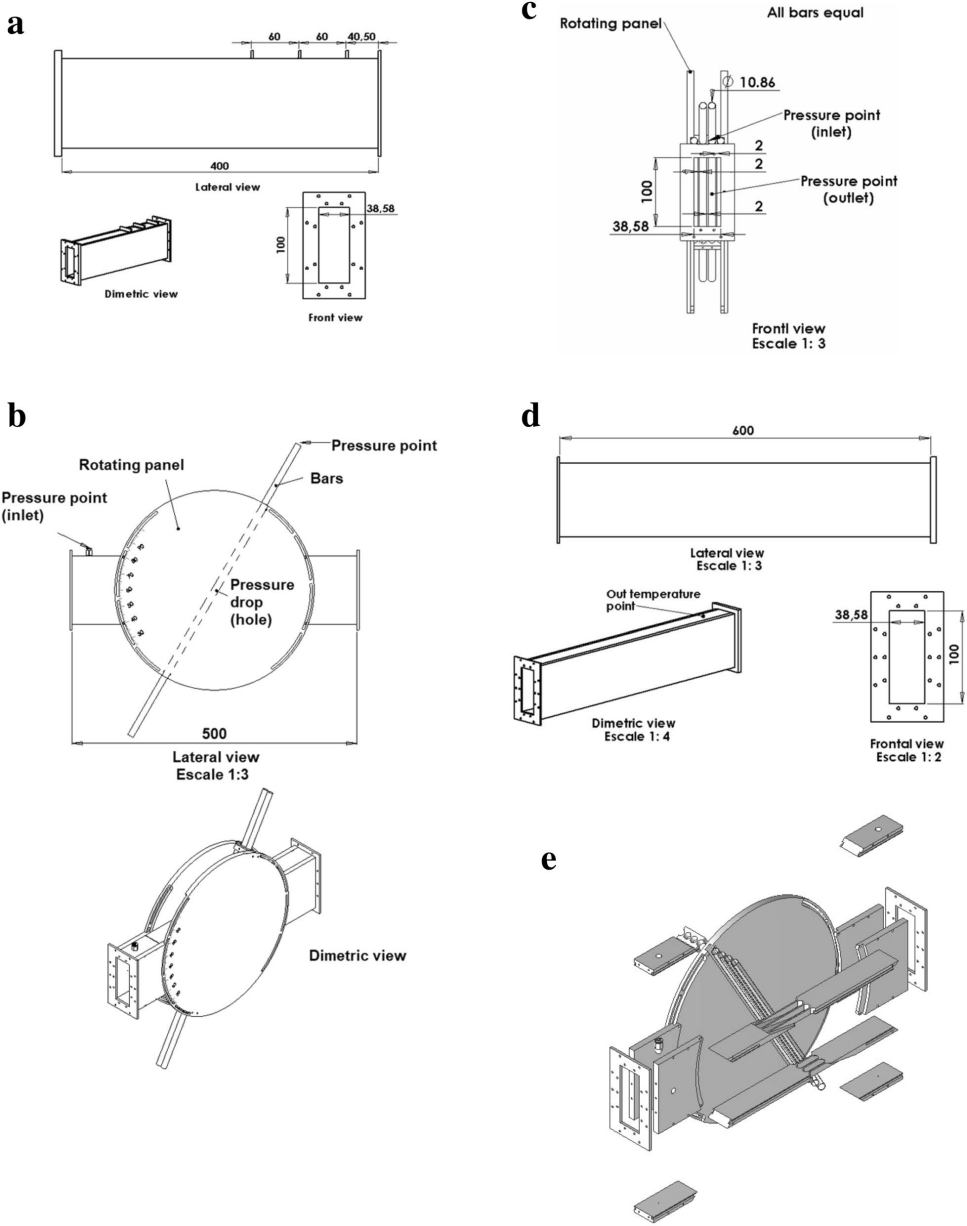
a Inlet section (lengths in mm). Created with Openscad 2021.01, openscad.org b. Main test section (lengths in mm). Created with Openscad 2021.01, openscad.org c Cross view of the main test section (lengths in mm). Created with Openscad 2021.01, openscad.org d Outlet section (lengths in mm). Created with Openscad 2021.01, openscad.org e Exploded view of the test section. Created with Openscad 2021.01, openscad.org.

The experimental setup consists of a rectangular test section that receives an air flow provided by an axial blower. The test section hosts a cell composed of two parallel central rods and two half rods embedded in the channel walls as shown in Fig. 1e, all of them having the same diameter. Figure 1a–e show the detailed geometry and dimensions of each part of the experimental setup. Figure 3 shows the flow setup.

Three sets of rods with different diameters are tested. Table 1 lists the geometric characteristics of each case. The rods are mounted on a protractor so that their angle respect to the flow direction can be varied between 90° and 30° (Figs. 1b and 3). All rods are made of stainless steel, and they are centered keeping the same gap distance between them. The relative position of the rods is fixed by two spacers located outside the test section.

**Table 1:** Rods’ diameter (*d*), gap between rods (*g*) for each arrangement. The tolerance is 0.05 mm.

*d* (mm)	*g* (mm)	*d*/*g*
10.86	1.99	5.45
8.30	4.55	1.82
6.50	6.35	1.02

**Figure 2:**
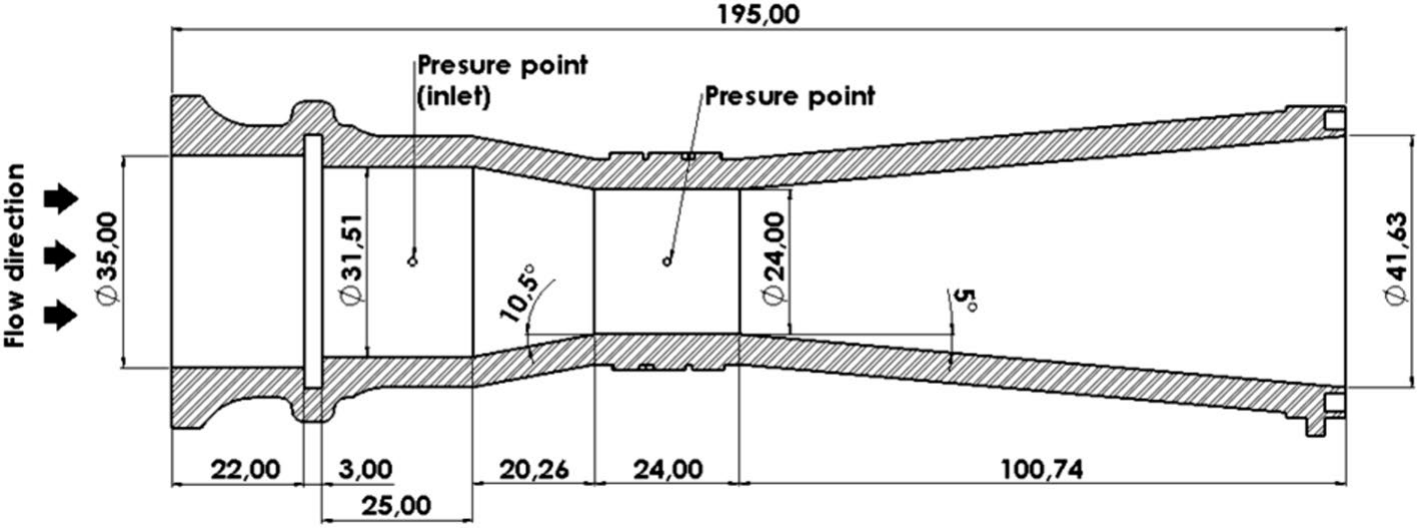
Diagram of the Venturi tube used to measure the inlet flow rate (lengths in mm). Created with Openscad 2021.01, openscad.org.

The inlet flow rate to the test section is measured by means of a calibrated Venturi Tube, shown in Fig. 2, which is monitored with a DP Cell Honeywell SCX. The fluid temperature at the exit of the test section is measured with a PT100 thermometer, and it is controlled at 45 ± 1 C°. To ensure a planar velocity profile and reduce turbulence levels at the channel inlet, the incoming flow is forced through three metallic screens. A settling distance of approximately 4 hydraulic diameters was taken between the last screen and the rods, whereas the outlet has a length of 11 hydraulic diameters.

**Figure 3:**
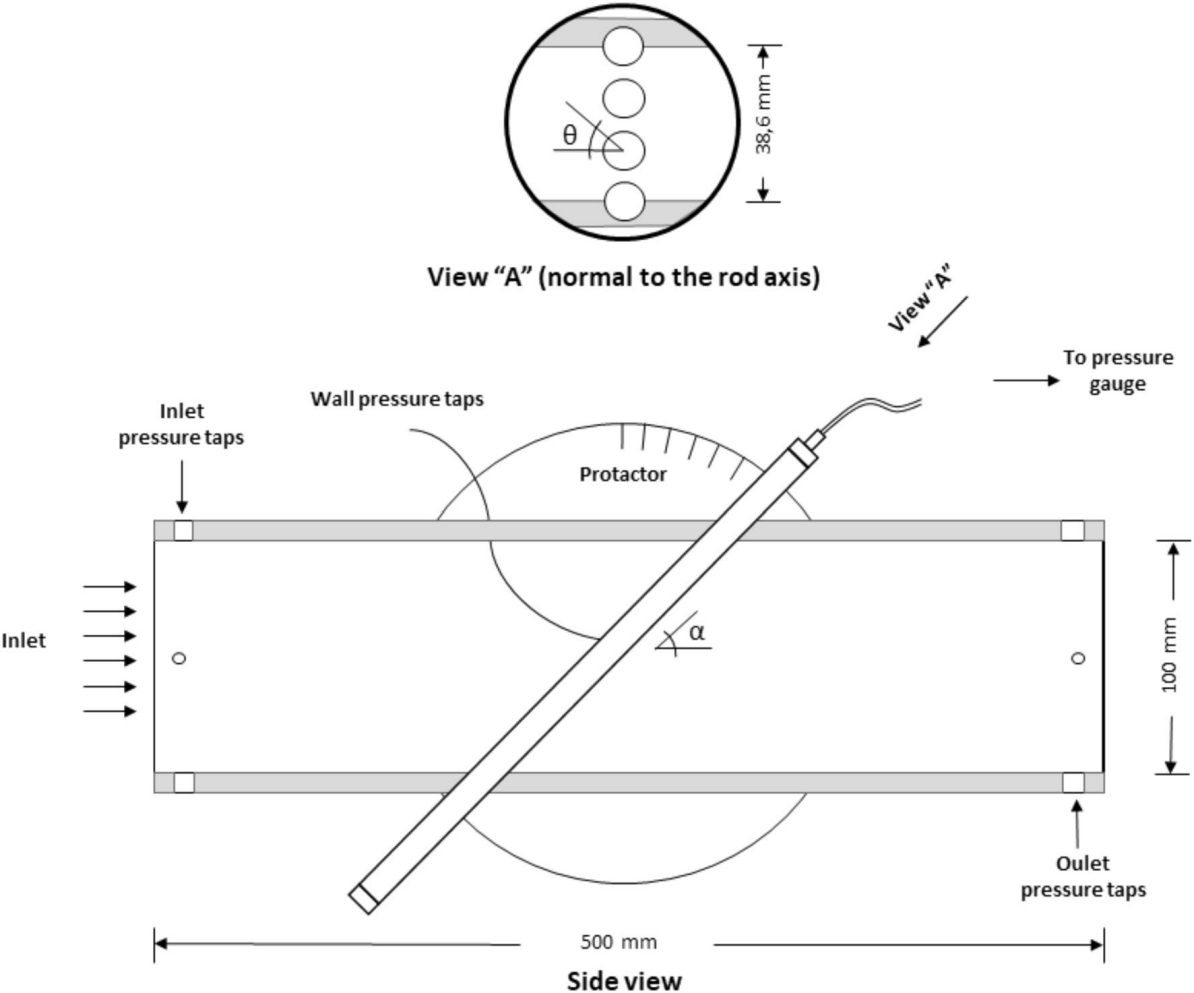
Flow setup. The channel walls are shown in grey. The flow goes from left to right and is obstructed by the rods. Note that the view “A” is normal to the rods axis. The external rods are semi-embedded in the lateral channel walls. The protractor is used to measure inclination angle $\alpha$. Created with Openscad 2021.01, openscad.org.

The pressure on the surface of one of the central rods is monitored through a 0.5-mm pressure tap at the middle plane of the test section. The pressure tap diameter corresponds to 5° angular span; hence the angular precision is approximately 2°. The monitored rod can be rotated around its axis, as can be seen in Fig. 3. The difference between the rod surface pressure and the pressure at the inlet of the test section was measured with a differential DP Cell Honeywell SCX Series. This pressure difference was measured for each arrangement of bars, varying the flow rate, the inclination angle, $\alpha$, and the azimuthal angle, $\theta$.

## Results

The goal of the experiment is measuring and interpreting the pressure drop between the channel inlet and the pressure on the surface of the central bar, for different azimuthal and inclination angles, $\theta$ and $\alpha$. In order to generalize the results, the pressure difference will be presented in dimensionless form as the Euler number:

$${Eu}_{w}\equiv \frac{{p}_{w}-{p}_{i}}{\frac{1}{2}\rho {u}_{i}^{2}} (1)$$

where $\rho$ is the fluid density, ${u}_{i}$ is the average inlet velocity, ${p}_{i}$ is the inlet pressure and and ${p}_{w}$ is the pressure at a given point of the rod wall. The inlet velocity was fixed within three different range levels determined by the opening of the inlet valve. The resulting velocities range between 6 and 10 m/s, corresponding to channel Reynolds numbers, $Re\equiv {u}_{i}H/\nu$ (where $H$ is the height of the channel and $\nu$ is the kinematic viscosity) between 40,000 and 67,000. The rod Reynolds numbers ($Re\equiv {u}_{i}d/\nu$) range between 2500 and 6500. The turbulent intensities estimated through the relative standard deviation of the signal recorded in the Venturi tube averages 5%.

**Figure 4:**
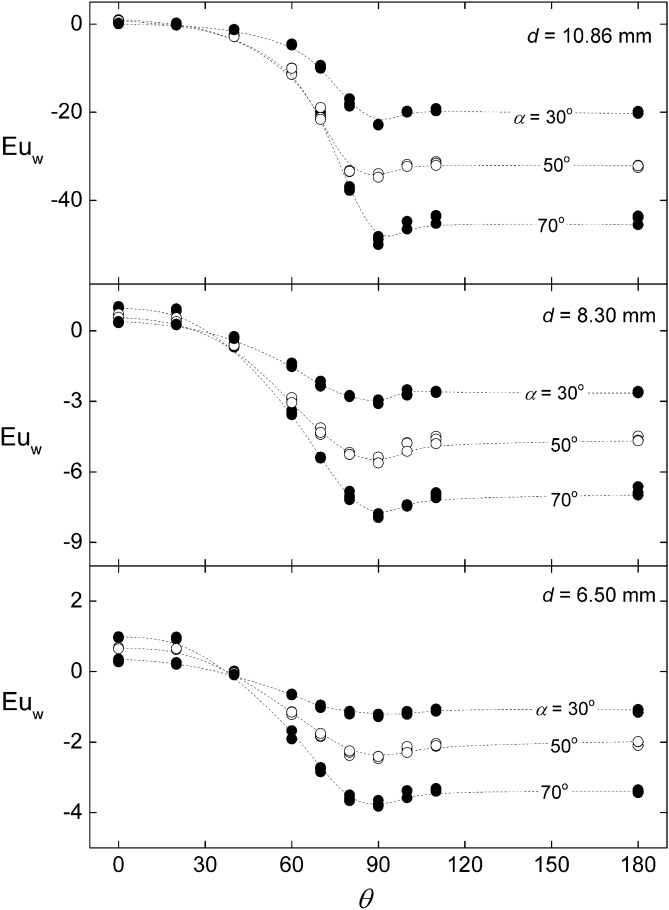
Variation of the wall Euler number around the rod for different inclinations angles and rod diameter. Created with Gnuplot 5.4, www.gnuplot.info.

Figure 4 shows the dependence of ${Eu}_{w}$ with the azimuthal angle $\theta$, parametrized by three inclinations, $\alpha$ = 30°, 50° and 70°. The measurements are separated in three graphics according to the diameter of the rods. It can be seen that the resulting Euler numbers, within the experimental uncertainties, are independent of the flow rate. The general dependence with *θ* follows the usual trend of the wall pressure about the perimeter of a round obstacle. At angles facing the flow, i.e. *θ* from 0 to 90°, the rod wall pressure decreases reaching a minimum value at 90°, corresponding to the gap between bars where the velocity is maximum due to the restriction of the flow area. Following, there is a pressure recovery for *θ* from 90° to 100°, and afterwards the pressure remains uniform due to the separation of the boundary layer at the back the rod wall. Notice that there is no shift of the angle of minimum pressure, indicating that possible interference from adjacent shear layers, like Coanda effect, are of secondary nature.

In what follows, we present an analysis of the results based on the hypothesis that the Euler number can be estimated solely by geometric parameters, namely, the characteristic length-ratios $d/g$ and $d/H$ (where $H$ is the height of the channel), and the inclination angle $\alpha$. A popular practical rule of thumb states that the fluid–structure forces on yawed rods are determined by the projection of the inlet velocity normal to the rods axis, ${u}_{n}={u}_{i}\mathrm{sin}\alpha$ . This is sometimes referred as the independence principle. One of the goals of the following analysis is to check whether this principle holds in our case, where flow and obstacles are confined inside a closed channel.

Let us consider the pressure measured at the front of the surface of the middle rod, i.e., *θ* = 0. According to the Bernoulli equation, the pressure at that position, ${p}_{o}$, satisfies:

2 $${p}_{o}-{p}_{i}=\frac{1}{2}\rho \left({u}_{i}^{2}-{u}_{o}^{2}\right)$$

where ${u}_{o}$ is the fluid velocity adjacent to the rod wall at *θ* = 0, and we are assuming that the irreversible losses are relatively small. Notice that the dynamic pressure is cast independently in the kinetic energy terms. If ${u}_{o}$ were null (i.e., stagnation conditions) the Euler number should be unity. However, it can be observed in Fig. 4 that at $\theta =0$ the resulting ${Eu}_{w}$ are close but not exactly equal to that value, particularly for larger inclinations. This suggests that the velocity on the rod surface at $\theta =0$ does not vanish, which can be chocked up to the upward deflection of the current lines produced by the inclination of the rods. Since the flow is confined at the top and the bottom of the test section, such deflection should generate a secondary recirculation that increases the axial velocity at the bottom and reduced the velocity at the top. Assuming that the magnitude of the mentioned deflection is the projection of the inlet velocity over the axis rod (*i.e.*, ${u}_{i}\mathrm{cos}\alpha$), the corresponding Euler number results:

3 $${Eu}_{o}\equiv \frac{{p}_{o}-{p}_{i}}{\frac{1}{2}\rho {u}_{i}^{2}}={\mathrm{sin}}^{2}\alpha $$

**Figure 5:**
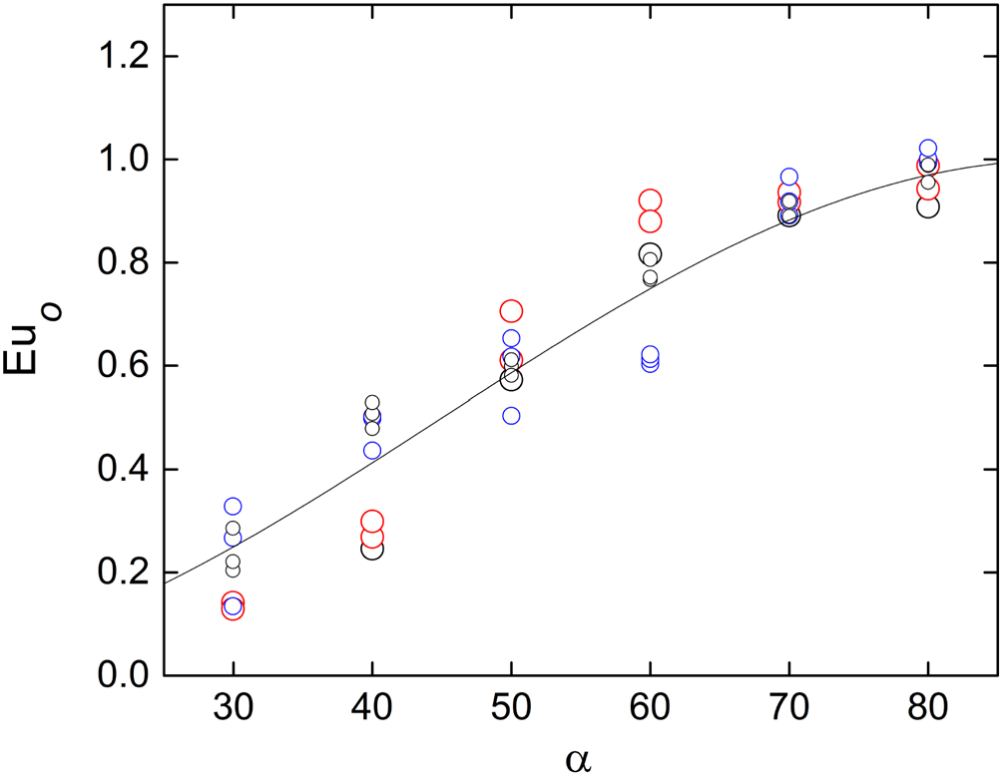
Variation of the wall Euler number at $\theta =0$ with the inclination angle. The curve corresponds to Eq. (3). Created with Gnuplot 5.4, www.gnuplot.info.

**Figure 6:**
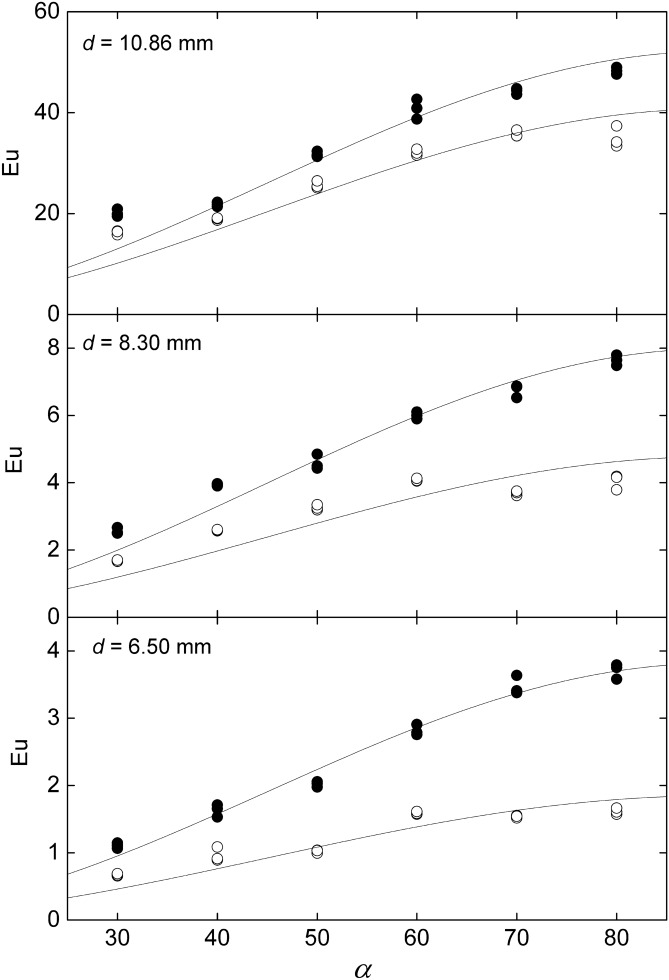
Variation of the wall Euler number, at $\theta =18{0}^{o}$ (full symbols) and the exit, (empty symbols) with the inclination angle. The curves corresponds to the independence principle, *i.e.*, $Eu\propto {\mathrm{sin}}^{2}\alpha$. Created with Gnuplot 5.4, www.gnuplot.info.

Figure 5 compares Eq. (3) with the corresponding experimental data, showing good agreement. The average deviation is 25% with 95% confidence level. Notice that Eq. (3) is in agreement with the independence principle. Likewise, Fig. 6 shows that the Euler numbers corresponding to the pressure on the surface at the back of the rod, ${p}_{180}$, and at the exit of the test section, ${p}_{e}$, also follows a trend proportional to ${\mathrm{sin}}^{2}\alpha$. However, in both cases, the coefficient depends on the rods diameter, which is reasonable since the latter determines the obstructed area. This feature is similar to the pressure drop across an orifice plate, where the flow passage is partially reduced at a certain location. In the present test section, the role of the orifice is played by the gap between bars. In such cases, the pressure experiences a substantial drop at the restriction and is partially recovered in the backward expansion. Viewing the restriction as a blockage in the direction normal to the rods axis, the pressure drop between the front and the back of the rods can be written as^18^:

4 $${p}_{o}-{p}_{180}={c}_{d} \left(\frac{{A}_{f}^{2}}{{A}_{m}^{2}}-1\right)\frac{1}{2}\rho {u}_{n}^{2}$$

where ${c}_{d}$ is a drag coefficient accounting for the partial pressure recovery between *θ* = 90° and *θ* = 180°, and ${A}_{m}$ and ${A}_{f}$ are the minimum and free cross section per unit length normal to the rod axis, which are related to the rod diameter by ${A}_{f}/{A}_{m}=\left(g+d\right)/g$. The corresponding Euler number is:

5 $${Eu}_{0-180}\equiv \frac{{p}_{o}-{p}_{180}}{\frac{1}{2}\rho {u}_{i}^{2}}= {c}_{d} \left(\frac{d}{g}+2\right)\frac{d}{g} {\mathrm{sin}}^{2}\alpha $$

**Figure 7:**
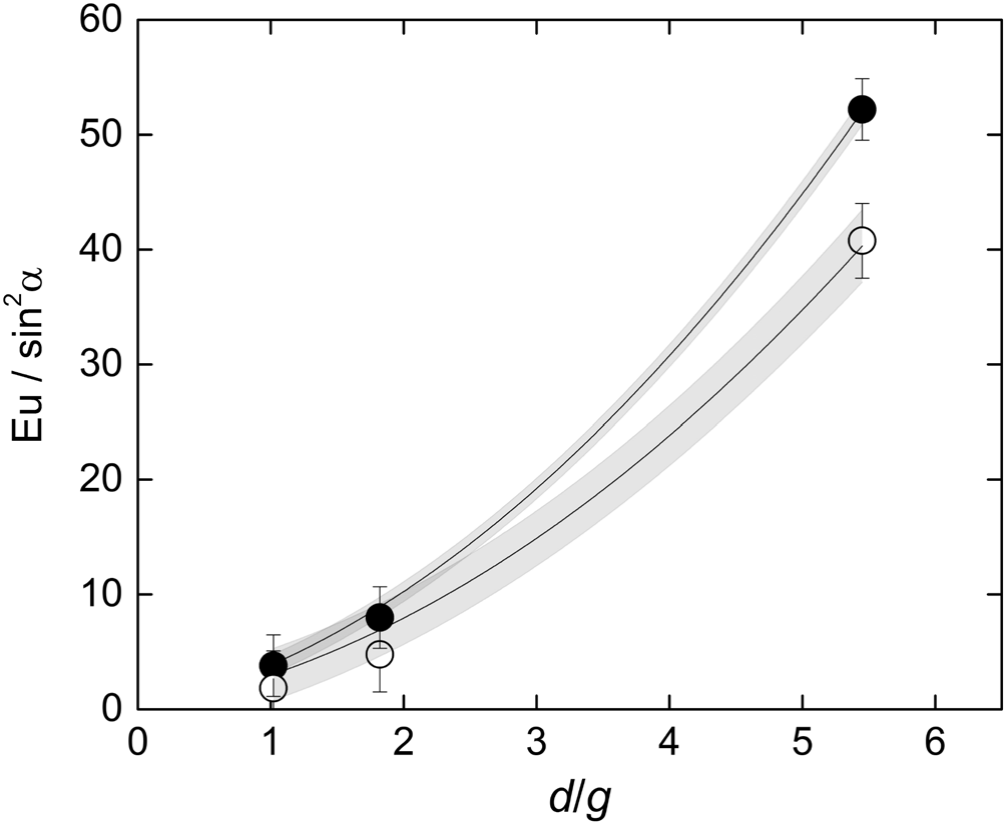
Dependence of the drag coefficient with $d/g$ for the pressure drop between the front and the back of the rod $\left({Eu}_{0-180}\right)$ and the total pressure drop between the channel inlet and outlet. The grey zones are the 67% confidence bands of the correlations. Created with Gnuplot 5.4, www.gnuplot.info.

Figure 7 shows the dependence of ${Eu}_{0-180}/{\mathrm{sin}}^{2}\alpha$ with $d/g$ showing excellent agreement with Eq. (5). The resulting drag coefficient is ${c}_{d}=1.28\pm 0.02$ with 67% confidence. Likewise, the same graphic also shows that the total pressure drop between the inlet and the outlet of the test section follows a similar trend, but with a different coefficient accounting for the pressure recovery in the backspace between the bars and the channel exit. The corresponding drag coefficient is ${c}_{d}=1.00\pm 0.05$ with 67% confidence.

### *Pressure on the rod surface at*$\theta ={90}^{o}$

The minimum pressure on the rod surface, ${p}_{90}$, at *θ* = 90°, requires a special treatment. According to the Bernoulli equation along a current line passing through the gap between bars, the pressure, ${p}_{g}$, and the velocity,${u}_{g}$, at the center of the gap between bars (which coincides with the middle point of the channel) are related by:

6 $${p}_{i}-{p}_{g}=\frac{1}{2}\rho \left({u}_{g}^{2}-{u}_{i}^{2}\right)$$

**Figure 8:**
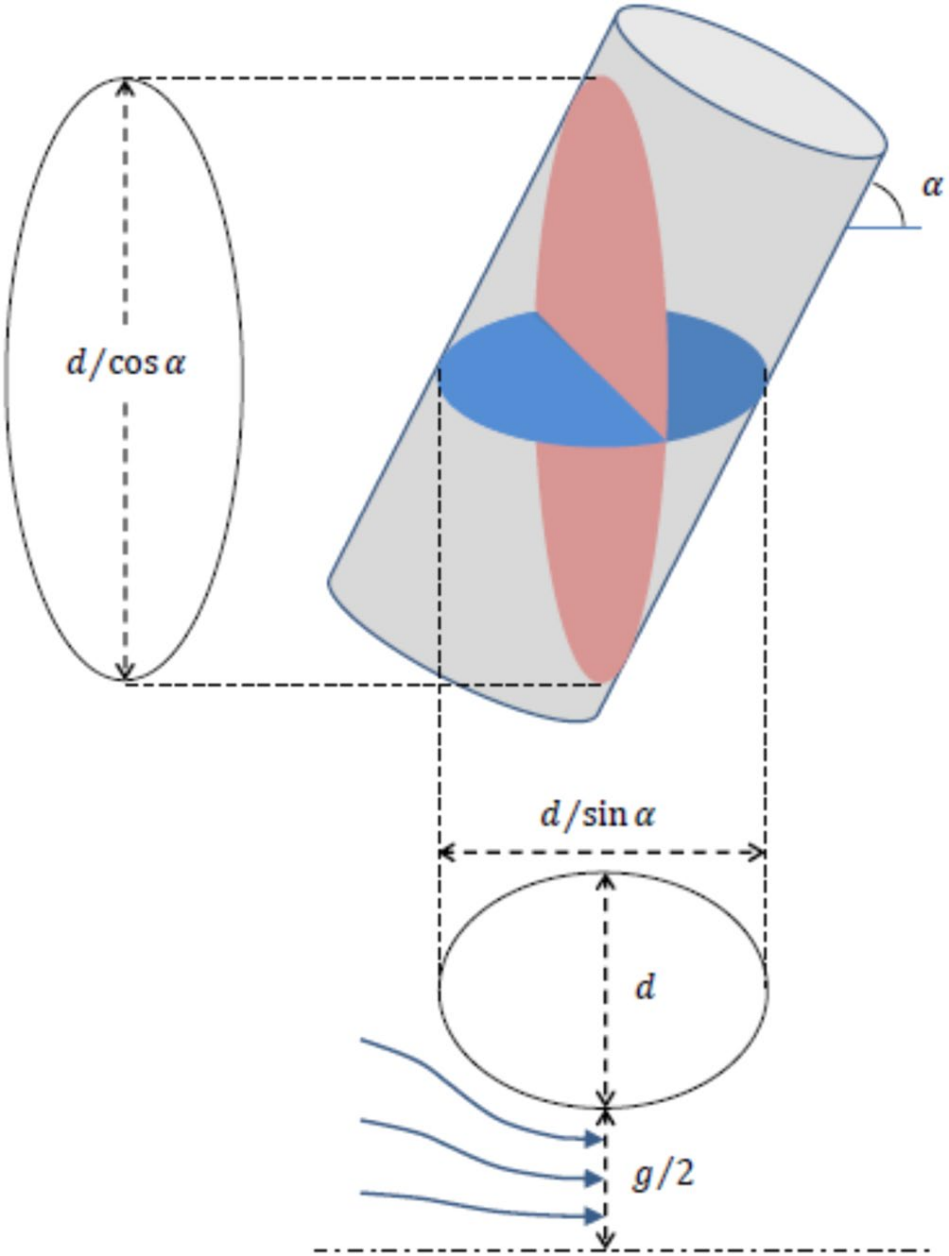
Diagram of the characteristic cross sections, frontal (left) and from above (below). Created with Microsoft Word 2019,

The pressure ${p}_{g}$ can be related to the rod surface pressure at *θ* = 90° by integrating the pressure profile across gap separating the central rods between the middle point and the wall (see Fig. 8). The balance of forces gives^19^:

7 $$\frac{dp}{dy}=\rho K\left(y\right) {u}_{g}^{2}$$

where $y$ is a coordinate that goes from the center point of the gap between the central rods perpendicularly to the rod surface, and $K$ is the curvature of the current line at position $y$. To produce an analytical assessment of the pressure on the rod surface, let us assume that ${u}_{g}$ is uniform and that $K\left(y\right)$ is linear. These assumptions were verified with numerical calculations. At the rod wall the curvature is determined by the ellipsoidal section of the bar at angle $\alpha$, namely, $K\left(g/2\right)=\left(2/d\right){\mathrm{sin}}^{2}\alpha$ (see Fig. 8). Then, regarding that due to the symmetry the curvature of the stream lines vanishes at $y=0$, the curvature at a generic coordinate $y$ is given by:

8 $$K=-\frac{4 {\mathrm{sin}}^{2}\alpha }{g d}y$$

Integrating Eq. (7) gives:

9 $${p}_{90}-{p}_{g}=-\frac{1}{2}\rho \left(\frac{g}{d}{\mathrm{sin}}^{2}\alpha \right){u}_{g}^{2}$$

Combining Eqs. (6) and (9), ${u}_{g}$ can be calculated as:

10 $${u}_{g}={u}_{i}\sqrt{\frac{1-{Eu}_{90}}{1+\frac{g}{d}{\mathrm{sin}}^{2}\alpha }}$$

where

11 $${Eu}_{90}\equiv \frac{{p}_{90}-{p}_{i}}{\frac{1}{2}\rho {u}_{i}^{2}}$$

On the other hand, by mass conservation, the average velocity $\langle {u}_{g}\rangle$ over the plane perpendicular to the flow at the measurement position is related to the inlet velocity as:

12 $$\langle {u}_{g}\rangle ={u}_{i}\frac{{A}_{i}}{{A}_{g}}$$

where ${A}_{i}$ is the cross-section flow area at the channel inlet, and ${A}_{g}$ is the cross-section flow area at the measurement position (see Fig. 8) given respectively by:

13 $${A}_{i}=3H\left(d+g\right)$$

14 $${A}_{g}={A}_{i}-\frac{3\pi }{4} \frac{{d}^{2}}{\mathrm{cos}\alpha }$$

**Figure 9:**
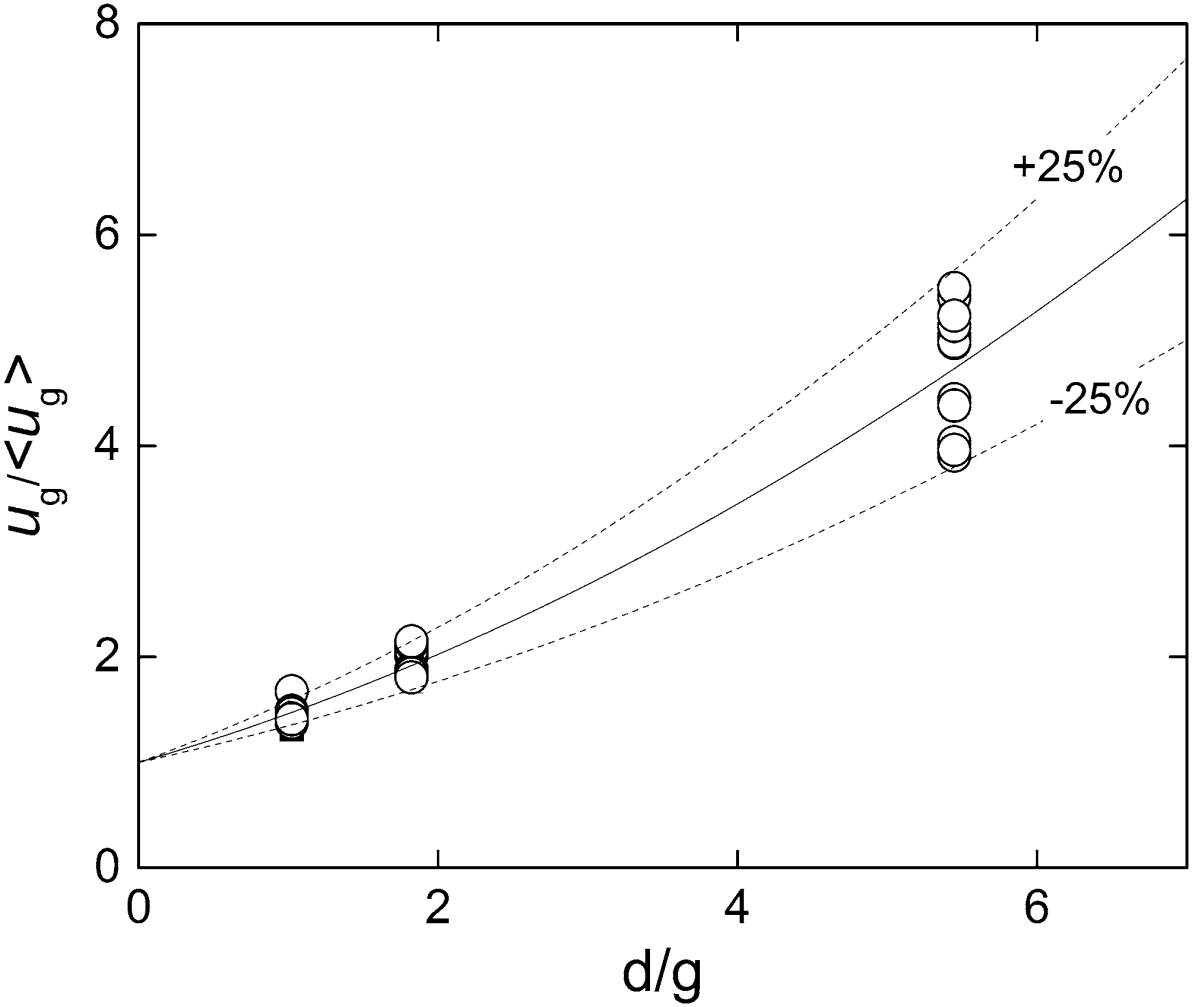
Ratio between the maximum ${u}_{g}$ and the average $\langle {u}_{g}\rangle$ velocities at the central cross section of the channel $.$ The solid and dashed curves corresponds to Eq. (5) and the bounds varying $\pm 25\%$ of the corresponding coefficients. Created with Gnuplot 5.4, www.gnuplot.info.

It should be noticed that ${u}_{g}$ is not equal to $\langle {u}_{g}\rangle$. In effect, Fig. 9 depicts the velocity ratio ${u}_{g}/\langle {u}_{g}\rangle$, calculated from Eqs. (10)–(14), plotted against the ratio $d/g$. Although there is some dispersion, it is possible to identify a trend, which was approximated by a second order polynomial:

15 $$\frac{{u}_{g}}{\langle {u}_{g}\rangle }\cong 1+0.41 \frac{d}{g}+0.05 {\left(\frac{d}{g}\right)}^{2}$$

Combining Eqs. (10) and (15) yields:

16 $${Eu}_{90}=1-{\Gamma }^{2}$$

where

17 $$\Gamma =\left[\frac{1+0.41 \frac{d}{g}+0.05 {\left(\frac{d}{g}\right)}^{2}}{1-\frac{\pi }{4} \frac{d}{H}\frac{1}{\left(1+\frac{g}{d}\right)\mathrm{cos}\alpha }}\right]\sqrt{1+\frac{g}{d}{\mathrm{sin}}^{2}\alpha }$$

**Figure 10:**
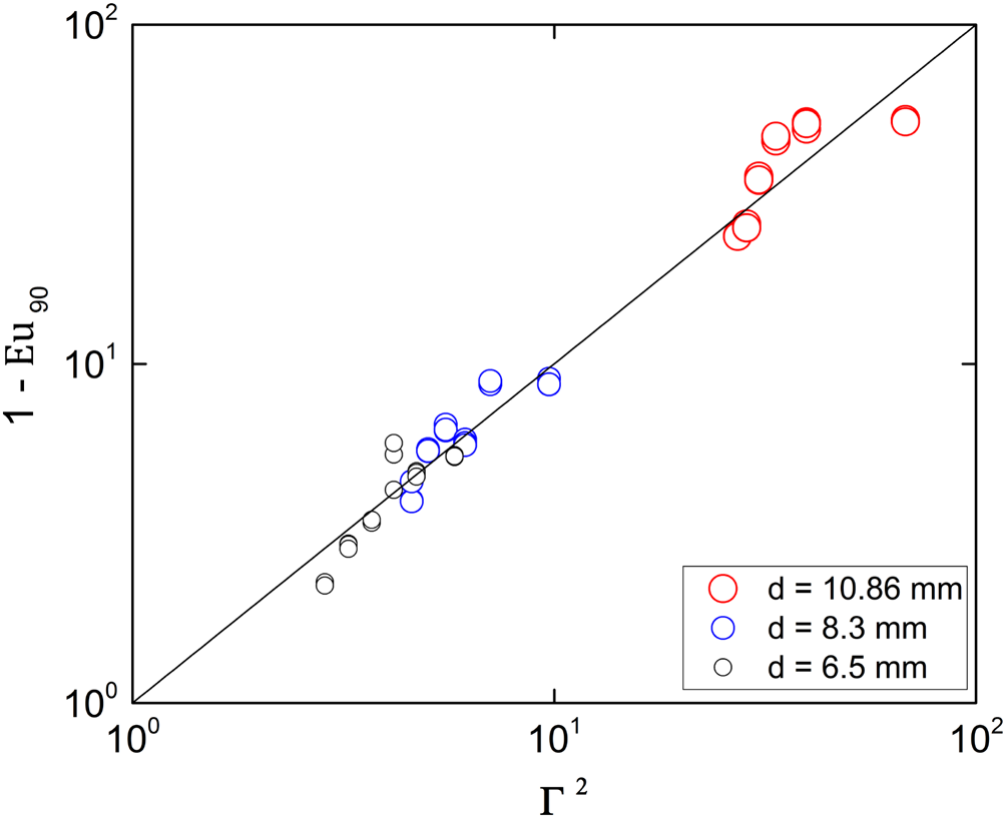
Wall Euler number at $\theta ={90}^{o}$. The curve corresponds to Eq. (16). Created with Gnuplot 5.4, www.gnuplot.info.

Figure 10 compares the experimental results for ${Eu}_{90}$ with Eq. (16). The average relative deviation is 25% with a confidence level of 95%.

### Net force acting on the rods

The net force, ${f}_{n}$, acting on the central rod perpendicular to its axis can be calculated by integrating the pressure over the rod surface, as:

18 $${f}_{n}=\frac{H}{\mathrm{sin}\alpha }\frac{d}{2} \oint {p}_{w}\,\mathrm{cos}\,\theta d\theta $$

where the first coefficient is the rod length inside the channel and the integral is performed between 0 and 2π.

The projection of ${f}_{n}$ on the stream direction should match the pressure force between the channel inlet and outlet, barring friction forces parallel to the rods and minor momentum flux imbalances due to incomplete profile development at the back. Accordingly,

19 $$3{f}_{n}\mathrm{sin}\alpha =3H\left(d+g\right)\left({p}_{i}-{p}_{e}\right)$$

The coefficient 3 in Eq. (19) accounts for the three rods.

In dimensionless form, we can then write:

20 $${Eu}_{\oint }=2\left(1+\frac{g}{d}\right){Eu}_{e}$$

where

21 $${Eu}_{\oint }\equiv \frac{\oint {p}_{w}\,\mathrm{cos}\,\theta d\theta }{\frac{1}{2}\rho {u}_{i}^{2}}$$

and

22 $${Eu}_{e}\equiv \frac{{p}_{i}-{p}_{e}}{\frac{1}{2}\rho {u}_{i}^{2}}$$

**Figure 11:**
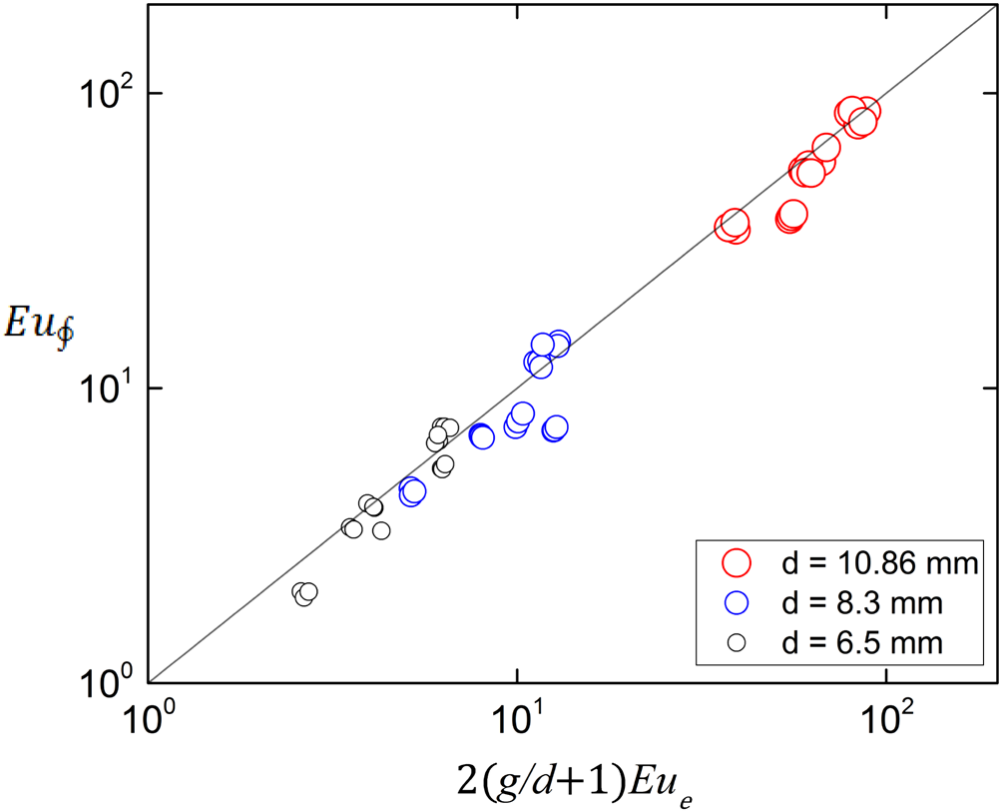
Balance of forces of the channel. The line corresponds to Eq. (20). The Pearson correlation coefficient is 0.97. Created with Gnuplot 5.4, www.gnuplot.info.

Figure 11 shows the plot of Eq. (20) for all the experimental conditions, showing good agreement. There is however a slight 8% bias to the right, which can be ascribed and used as an estimation of the momentum imbalance between the channel inlet and the outlet.

## Conclusions

The pressure at the rod surface wall and the pressure drop in a channel with a transverse line of four inclined cylindrical rods were measured varying the inclination angle of the rods. Three assemblies of rods with different diameters were tested. Within the range of Reynolds numbers of the test, between 2500 and 6500, the Euler numbers are independent of the flow rate. The pressure on the surface of the central rod follows the usual trend observed in cylinders, with the maximum at the front, the minimum at the lateral gap between bars, and the partial recovery at the back due to the detachment of the boundary layer.

The experimental data were analyzed using momentum conservation considerations and semi-empirical assessments, searching for invariant dimensionless numbers that relate the Euler numbers with the characteristic dimensions of the channel and rods. All the geometrical characteristics of the blockage are fully represented by the ratios between the rods diameter and the gap between rods (in the lateral direction) and the channel height (in the vertical direction).

It was found that the independence principle holds for most of the Euler numbers characterizing the pressure at different locations, that is, the group is independent of the inclination angle provided that the inlet velocity projection normal to the rods is used to non-dimensionalize the pressure. Moreover, it was shown that this feature is consistent with the mass and momentum conservation equations of the flow, which supports the mentioned empirical principle. Only the pressure on the rod surface at the gap between rods presents a slight deviation from this principle. Dimensionless semi-empirical correlations were produced that can be useful for designing similar hydraulic units. This classical methodology is in line with similar recently reported applications of the Bernoulli equation in hydraulics and hemodynamics^20,^^21,^^22,^^23,^^24^.

A particularly interesting result stems from the analysis of the pressure drop between the inlet and outlet of the test section. Within the experimental uncertainties, the resulting drag coefficient equals unity, which suggests the existence of the following invariant parameter:

23 $$\frac{{Eu}_{e}}{ \left(\frac{d}{g}+2\right)\frac{d}{g} {\mathrm{sin}}^{2}\alpha }\approx 1$$

Notice that the magnitude $\left(d/g+2\right)d/g$ in the denominator of Eq. (23) is the magnitude within the parenthesis in Eq. (4), which otherwise can be calculated with the minimum and free cross section normal to the rods, ${A}_{m}$ and ${A}_{f}$. This suggests the conjecture that this invariant parameter might hold generally in channels obstructed by other yawed obstacles, like non-circular rods or grids, provided that the Reynolds number remains within the range of the current study (40,000–67,000 for the channel and 2500–6500 for the rods). It should be noted that if there are temperature differences inside the channel, the fluid density might be affected. In such cases, the relative variation of the Euler number can be assessed by the thermal expansion coefficient multiplied by the maximum expected temperature difference.

## Data Availability

All data generated or analyzed during this study are included in this published article.
